# Epidemiology and zoonotic transmission of *mcr*-positive and carbapenemase-producing Enterobacterales on German turkey farms

**DOI:** 10.3389/fmicb.2023.1183984

**Published:** 2023-06-02

**Authors:** Katja Nordhoff, Martina Scharlach, Natalie Effelsberg, Carolin Knorr, Dagmar Rocker, Katja Claussen, Richard Egelkamp, Alexander C. Mellmann, Andreas Moss, Ilona Müller, Sarah Andrea Roth, Christiane Werckenthin, Anne Wöhlke, Joachim Ehlers, Robin Köck

**Affiliations:** ^1^Lower Saxony State Office for Consumer Protection and Food Safety (LAVES), Oldenburg, Germany; ^2^Perioperative Inflammation and Infection, Department of Human Medicine, Carl von Ossietzky University of Oldenburg, Oldenburg, Germany; ^3^Public Health Agency of Lower Saxony (NLGA), Hanover, Germany; ^4^Institute of Hygiene, University Hospital Münster, Münster, Germany; ^5^Hygiene and Environmental Medicine, University Medicine Essen, Essen, Germany

**Keywords:** colistin resistance (*mcr*), zoonosis, one health, poultry, farmers, personnel, staff

## Abstract

**Introduction:**

The emergence of carbapenem-resistant bacteria causing serious infections may lead to more frequent use of previously abandoned antibiotics like colistin. However, mobile colistin resistance genes (*mcr*) can jeopardise its effectiveness in both human and veterinary medicine. In Germany, turkeys have been identified as the food-producing animal most likely to harbour *mcr*-positive colistin-resistant Enterobacterales (*mcr*-Col-E). Therefore, the aim of the present study was to assess the prevalence of both *mcr*-Col-E and carbapenemase-producing Enterobacterales (CPE) in German turkey herds and humans in contact with these herds.

**Methods:**

In 2018 and 2019, 175 environmental (boot swabs of turkey faeces) and 46 human stool samples were analysed using a combination of enrichment-based culture, PCR, core genome multilocus sequence typing (cgMLST) and plasmid typing.

**Results:**

*mcr*-Col-E were detected in 123 of the 175 turkey farms in this study (70.3%). *mcr*-Col-E isolates were *Escherichia coli* (98.4%) and *Klebsiella* spp. (1.6%). Herds that had been treated with colistin were more likely to harbour *mcr*-Col-E, with 82.2% compared to 66.2% in untreated herds (*p* = 0.0298). Prevalence also depended on husbandry, with 7.1% *mcr*-Col-E in organic farms compared to 74.5% in conventional ones (*p* < 0.001). In addition, four of the 46 (8.7%) human participants were colonised with *mcr*-Col-E. *mcr*-Col-E isolates from stables had minimum inhibitory concentrations (MICs) from 4 to ≥ 32 mg/l, human isolates ranged from 4 to 8 mg/l. cgMLST showed no clonal transmission of isolates. For one farm, plasmid typing revealed great similarities between plasmids from an environmental and a human sample. No CPE were found in turkey herds or humans.

**Discussion:**

These findings confirm that *mcr*-Col-E-prevalence is high in turkey farms, but no evidence of direct zoonotic transmission of clonal *mcr*-Col-E strains was found. However, the results indicate that plasmids may be transmitted between *E. coli* isolates from animals and humans.

## Introduction

Infections with multidrug-resistant bacteria are a major health burden in both human and veterinary medicine. Selective pressure from antimicrobial use in human and animal populations fuels the development of antibiotic resistance (European Food Safety Authority, and European Centre for Disease Prevention and Control, 2022).

The incidence of human infections caused by multidrug-resistant organisms, particularly carbapenem-resistant bacteria such as *Klebsiella pneumoniae* and *Acinetobacter baumannii*, is increasing in Germany (Robert Koch-Institut, 2022). This has prompted a need for alternative therapeutic options. Consequently, the antibiotic colistin has been reintroduced as a last resort treatment option for infections caused by carbapenem-resistant bacteria (Falagas and Kasiakou, 2005). In Germany, systemic colistin therapy was (re-) approved in 2012, in spite of severe adverse effects such as neurotoxicity and nephrotoxicity (Yahav et al., 2012). In 2016, the WHO listed colistin as one of the critically important antimicrobial drugs for human therapy in accordance with the following criteria: colistin is one of a limited number of treatment options in serious bacterial infections, and the bacteria, as well as their resistance determinants, can be transmitted to humans from non-human sources (World Health Organization, 2005). For the same reasons, carbapenems have been prioritised in this group from the first draft of the report (World Health Organization, 2017).

Resistance to colistin is not a new phenomenon: bacteria such as *Proteus* spp. have long been known to be intrinsically resistant (Olaitan et al., 2014). While intrinsic resistance should certainly not be underestimated, the zoonotic transmission of bacteria and their resistance genes between humans and animals or vice versa has been a much greater One Health concern in recent years (Hernando-Amado et al., 2019). Such transmission has been shown to be likely for extended-spectrum β-lactamase (ESBL) producing (Fischer et al., 2017), carbapenemase-producing (CPE) (Elmonir et al., 2021), and colistin-resistant Enterobacterales (Col-E) (Nakano et al., 2021; Viñes et al., 2021).

The discovery of the plasmid-mediated *mcr*-*1* (mobile colistin resistance) gene has added a new dimension to colistin resistance of gram-negative bacteria both in livestock primary production and human medicine: such newly discovered plasmids could drastically accelerate zoonotic transmission (Liu et al., 2016). To date, 10 MCR-family genes and their variants (*mcr-1* to *mcr-10*) have been identified in various bacteria from humans, animals, food, farms and the environment (Hussein et al., 2021).

On the veterinary side, orally administered colistin is widely used to treat enteric infections in food-producing animals because of its lack of systemic resorption (Catry et al., 2015). In Germany, 42% of the colistin used for veterinary purposes is used for the treatment of poultry (Kietzmann et al., 2019). An increasing prevalence of colistin-resistant gram-negative bacteria in turkeys would be a particular cause for concern, as these animals are most often treated as a group rather than individually. This raises the risk of horizontal transmission of resistance traits among them. Indeed, among livestock and food, Col-E are most common in samples from turkeys (Irrgang et al., 2016; Alba et al., 2018; Zając et al., 2019).

Although carbapenems are not licensed for use in veterinary medicine, carbapenemase-producing bacteria are part of the panel investigated for the annual national monitoring programme in the European Union that covers the entire food chain, and CPE isolates have occasionally been identified in livestock (Fischer et al., 2012, 2013, 2017; Irrgang et al., 2017, 2020).

According to the German national as well as regional surveillance systems for antimicrobial resistance in clinical isolates from humans, resistance of *Escherichia coli* to imipenem or meropenem was <0.1% between 2008 and 2020. In the same period, resistance of *K. pneumoniae* to these drugs increased from <0.1 to 0.5 and 0.3%, respectively (Niedersächsisches Landesgesundheitsamt, 2021; Robert Koch-Institut, 2022). For human medicine, data on resistance to colistin in Germany are rare because Enterobacterales are not routinely tested.

At a methodological level, using conventional culture-based techniques focusing on commensal *E. coli* collected for screening purposes to detect colistin- and carbapenem-resistant Enterobacterales runs the risk of underestimating their prevalence. Enrichment followed by resistance gene pre-screening by PCR is far more sensitive (Irrgang et al., 2019) and sets the stage for sequencing approaches.

This study used this more sensitive combination of methods to investigate the prevalence of Col-E and CPE not only in German turkeys but also in their human contacts. Identical methods were applied to samples from animal faeces and human stool to compare isolates and their genomic profiles by means of microbiological, PCR-based and sequencing strategies.

## Methods

### Studied populations and sample collection

Between March 2018 and February 2019, local veterinary authorities visited 175 turkey farms in connection with the annual official zoonosis monitoring programme of the European Union based on Directive 2003/99/EC. The programme is designed to include a number of farms that allows statistically significant conclusions at a national level. This number is then divided among the German federal states depending on the percentage of animals kept in each state, making Lower Saxony the largest contributor for both farm- and slaughter-related data on turkeys. The farms in this study kept a minimum of 500 animals per fattening period. They were distributed in the northwestern part of Lower Saxony, Germany. As per the programme guidelines, sampling took place during the 3 weeks before the animals’ transport to the slaughterhouse. The standard official sampling form already contained a field for the husbandry system. For the purpose of this study, the form was supplemented by additional tick boxes to record if and how many times the herd had been treated with colistin. For sampling, two pairs of boot swabs were taken for each farm [i.e., boot swabs were put on the boots and the sample was taken by walking around in the poultry house, ensuring that all sections in a house were represented in a proportionate way (European Commission, 2012)]. The faecal material adherent to the boot swabs was then processed as described below.

During their visits to the farms, staff of the local veterinary authorities acquainted farmers, their employees and relatives with the study. They handed out test kits containing equipment for a self-sampling of stool swabs together with written and pictured instructions for sampling, as well as an informed consent form. In addition, participants were asked to fill in a questionnaire to analyse potential risk factors for colonisation with *mcr*-Col-E. Questions referred to the kind and duration of contact with poultry, contact with other livestock, consumption of antibiotics, hospitalisation, work in medical care and travel. Approval by the Ethics Committee of the University of Münster (No. 2018-008-f-S) had been obtained in advance.

### Molecular and culture-based methods targeting *mcr*-positive colistin-resistant Enterobacterales (*mcr*-Col-E)

A harmonised protocol was used to analyse samples from both animal and human sources, although some minor adjustments were required to meet accreditation requirements. Any diagnostic modifications made for the human stool samples are indicated in square brackets as appropriate. For colistin susceptibility testing, faecal material from boot swabs and human stool samples was dissolved in 1–2 ml (depending on the amount of material) of buffered peptone water and mixed thoroughly. Of this mixture, 250 μl were added to 3 ml of buffered peptone water containing 2 mg/l colistin and incubated for 18–22 h at 37 ± 1°C [36 ± 1°C] aerobically. An aliquot of 200 μl was then used for DNA extraction before screening for *mcr-1* as well as *mcr-2* genes with a multiplex PCR method (Cavaco et al., 2016). Samples with positive or ambiguous PCR results were further analysed, streaking 10 μl [1 μl] on chromID® Colistin R (bioMérieux, Marcy-l’Etoile, France), Super Polymixin (ELITechGroup, Puteaux, France) and MacConkey agar (CLED agar, both BD, Franklin Lakes, NJ, United States), respectively. After incubation of the agar plates as described above, one colony of *mcr-*Col-E was selected at random and analysed with MALDI-TOF mass spectrometry (MS) (MALDI biotyper®, Bruker Daltonic, Bremen, Germany) [VITEK®2 Compact, bioMérieux] to confirm the species identification. The colony was then subcultured on Columbia blood agar with a disk containing 10 μg colistin to maintain selection pressure (Oxoid by Thermo Fischer Scientific, Wesel, Germany). To verify the supposed *mcr* gene(s) in the respective isolates, the PCR method indicated above was applied to all isolates, control strains (*mcr-1*: *E. coli* NCTC 13846*, mcr-2: E. coli* RKI 278/17) and negative control (water ad iniectabilia, Braun, Melsungen, Germany) respectively. During the course of the study, additional methods became available. Therefore, 141 isolates of animal origin that had not been analysed at this point were also subjected to a multiplex PCR for *mcr-1* to *mcr-5* (Rebelo et al., 2018). All isolates were stored at −80°C (CRYOBANK®, Mast, Bootle, United Kingdom).

### Molecular and culture-based methods targeting CPE

The boot swabs were soaked in 450 ml of buffered peptone water and incubated as indicated above. Additionally, 10 μl were streaked onto ChromID® OXA-48 agar and chromID® CARBA agar (both bioMérieux), respectively. Incubation followed the instructions of the manufacturer.

Overnight cultures of boot swab and human faecal samples and aliquots of the stool suspensions were mixed with glycerol (1:3) and stored at −20°C until further processing. For genotypic detection of CPE, samples were thawed and 100 μl of each aliquot was transferred into a vial with 9 ml buffered peptone water (ReadyTube™ 9 bpW, Merck, Darmstadt, Germany) with 50 mg/l vancomycin and 0.25 mg/l ertapenem and incubated overnight at 37 ± 1°C. From each enrichment, 200 μl were transferred into a 1.5 ml reaction tube. Up to five samples were pooled in one tube and centrifuged at 13,400 rpm for 5 min. DNA was extracted from the pellets using the DNeasy® Blood & Tissue Kit (Qiagen, Hilden, Germany) following the manufacturer’s protocol for ‘Purification of total DNA from Animal Tissue.’ DNA was stored at 4°C for <24 h or at −20°C for later use. Presence of carbapenemase genes *bla*_NDM_, *bla*_KPC_, *bla*_VIM_, *bla*_IMP_ and *bla*_OXA-48_ in the pooled samples was checked in an RT-PCR using the Check-Direct CPE Kit (Check-Points B.V., Wageningen, Netherlands). In case of a positive pool sample, individual samples were tested with conventional PCRs to confirm the result. DNA from individual samples was extracted as described above. A set of 5 PCRs was run for each sample using the primers indicated in Table 1. All PCRs were run using the following protocol: Initial denaturation at 94°C for 5 min, followed by 30 cycles of 94°C for 1 min, 56°C for 30, 72°C for 1 min and a final elongation for 7 min at 72°C. PCR products were checked on gel (amplicon size see Table 1). Isolates from PCR-positive samples were cultured on ChromID® Carba and ChromID® OXA-48 (both bioMérieux) as well as MacConkey plates. Species identification was done by MALDI-TOF MS (Microflex® LT, Bruker Daltonik).

**Table 1:** Primers and amplicon size for CPE-PCR analysis.

Gene	Forward primer	Reverse primer	Product length	Reference
*bla*_NDM_	GGG CAG TCG CTT CCA ACG GT	GTA GTG CTC AGT GTC GGC AT	475 bp	Mushtaq et al. (2011)
*bla*_KPC_	ATG TCA CTG TAT CGC CGT CT	TTT TCA GAG CCT TAC TGC CC	950 bp	Schechner et al. (2009)
*bla*_VIM_	GAT GGT GTT TGG TCG CAT A	CGA ATG CGC AGC ACC AG	390 bp	Ellington et al. (2007)
*bla*_IMP_	GGA ATA GAG TGG CTT AAY TCT C	CCA AAC YAC TAS GTT ATC T	188 bp	Ellington et al. (2007)
*bla*_OXA-48_	TTG GTG GCA TCG ATT ATC GG	GAG CAC TTC TTT TGT GAT GGC	810 bp	Poirel et al. (2004)

### Determination of antimicrobial susceptibility

For the determination of colistin minimum inhibitory concentrations (MICs), a commercially available kit (ComASP® Colistin, bestbion, Cologne, Germany) was used according to the manufacturer’s instructions.

### Molecular typing of isolates and plasmids

*mcr-1*-positive *E. coli* isolates from stool samples together with isolates from corresponding and additional randomly selected farms were used for molecular typing. As described above, any diagnostic modifications made for the human stool samples are indicated in square brackets as appropriate.

Swabs from each isolate were streaked onto Columbia agar plates containing 5% sheep blood (Oxoid by Thermo Fischer Scientific). Each agar plate was then equipped with a colistin disc (Oxoid by Thermo Fischer Scientific) and incubated at 37 ± 1°C for 24 h. After that, colonies nearest to the colistin disc were transferred into buffered peptone water containing 2 mg/l colistin and incubated at 37 ± 1°C for 24 h.

1.5 ml of these cultures were used for DNA extraction. Genomic DNA of *E. coli* isolates was extracted with the Maxwell® RSC Instrument (an automated nucleic acid purification platform) using the Maxwell® RSC Whole Blood DNA Kit (Promega, Walldorf, Germany) [smart DNA prep kit (Analytik Jena, Jena, Germany)] according to the manufacturer’s instructions. Quantus™ fluorometer was used to measure the concentration of genomic DNA following the manufacturer’s instructions (Promega, Walldorf, Germany).

Sequencing libraries were prepared using the Illumina® Nextera™ DNA Flex Library Preparation Kit and Nextera™ DNA CD Indexes (96 Indexes, 96 Samples) according to the manufacturer’s instructions (Illumina Inc., San Diego, CA, United States). The normalised and pooled DNA libraries were loaded onto the flow cell for sequencing. Sequencing (2 × 250 bp paired-ends) [2 × 150 bp paired-ends] was performed on a MiSeq using the Illumina 500 [300] cycles V2 MiSeq reagent kit (Illumina Inc.).

For genotyping of isolates, raw sequence data were assembled with SKESA v2.3 and allelic profiles created using a task template based on 2,513 cgMLST targets of *E. coli* [based on core genome MLST scheme from EnteroBase^1^ in Ridom SeqSphere^+^ version 8.2.0 (2021–12) (Ridom GmbH, Münster, Germany)]. A minimum spanning tree was created from these profiles using the ‘Pairwise ignore missing values’ option in SeqSphere^+^.

For analysis of plasmids, genomic DNA (gDNA) was extracted using the NEB Monarch® Genomic Purification Kit (New England Biolabs, Ipswich, MA, United States). Isolates were sequenced on a PacBio Sequel IIe system (Pacific Biosciences. Menlo Park, CA, United States) using a 20 kb insert size library and the SMRTbell® Express Template Prep Kit 2.0. Raw sequences were *de novo* assembled using the hierarchical genome assembly process (HGAP) and analysed using the SMRT®Link software suite v8 with default parameters for microbial assembly. Final assembly contigs were extracted in FASTA format.

The final assembly files were uploaded to ResFinder v3.2 (Zankari et al., 2012) to determine the contig containing the *mcr* gene. The extracted sequences of the respective contigs were uploaded to PlasmidFinder v2.1 (Carattoli et al., 2014) to predict the respective plasmid replicon type. Plasmid sequences were annotated using a local installation of the NCBI Prokaryotic Genome Annotation Pipeline (PGAP) v4.10 (Tatusova et al., 2016). Annotated sequences were aligned and gene content around the *mcr* locus was compared in Mauve v20150226 (Darling et al., 2004). Raw and assembled sequence data have been deposited under the NCBI BioSample number PRJNA934726.

### Analysis and statistics

For analysis and visualisation of data, R (R Core Team, 2021), RStudio (RStudio Team, 2021) and the packages tidyverse (Wickham et al., 2019), here (Müller and Bryan, 2021), janitor (Firke, 2021), AMR (Berends et al., 2022), ggprism (Dawson, 2021) and patchwork (Petersen, 2021) were used. Considering the hypothesis that the use of antibiotics leads to higher resistance rates, a one-tailed Fisher’s exact test from rstatix (Kassambara, 2021) was applied to test the association between *mcr*-Col-E carriage, colistin treatment and husbandry system. *p* < 0.05 was considered significant. Due to the limited number of samples, no such calculations were performed for the data relating to the human participants.

## Results

### Epidemiology

The veterinary authorities visited 175 turkey farms. One-hundred fifty-three (87.4%) of these used conventional farming systems, 14 (8.0%) were organic farms, and for eight (4.6%) there was no data on husbandry. Regarding medication, 130 of the 175 herds (74.3%) had not been treated with colistin during the fattening period. Of the 45 herds (25.7%, all conventional) where colistin had been administered, 35 (20.0%) had received one course of treatment, eight (4.6%) had been treated twice, one (0.6%) herd received three courses of treatment, and one (0.6%) more than three. There are no data on the use of antibiotics other than colistin.

For the collection of human samples, 209 test kits were handed out on 126 farms. Forty-six (22%) of these, originating from 31 farms, were returned and fulfilled the inclusion criteria (stool sample, fully completed questionnaire and informed consent).

Table 2 shows that participants were mostly farmers and their relatives. Their ages ranged from 14 to 79 years. Most of them were male (31, 67.4%). Twenty (43.5%) participants reported contact with livestock other than poultry. Ten persons reported having taken antibiotics, four (8.7%) had been hospitalised (solely in Germany); two (4.3%) participants worked in medical care and 13 (28.3%) reported having travelled abroad (unspecified where), all within the last 12 months.

**Table 2:** Participants and their reported risk factors differentiated by colonisation with *mcr*-Col-E (*n* = 46, multiple risk factors possible).

	*mcr*-Col-E	
	yes	no
Farmer	2	22
Staff	0	7
Family member	2	12
Unknown	0	1
Risk factors		
Other livestock contact	0	20
Antibiotics	0	10
Hospitalisation	0	4
Working in medical care	0	2
Travelling abroad	1	12

### *mcr* genes and colistin-resistant Enterobacterales

**Figure 1:**
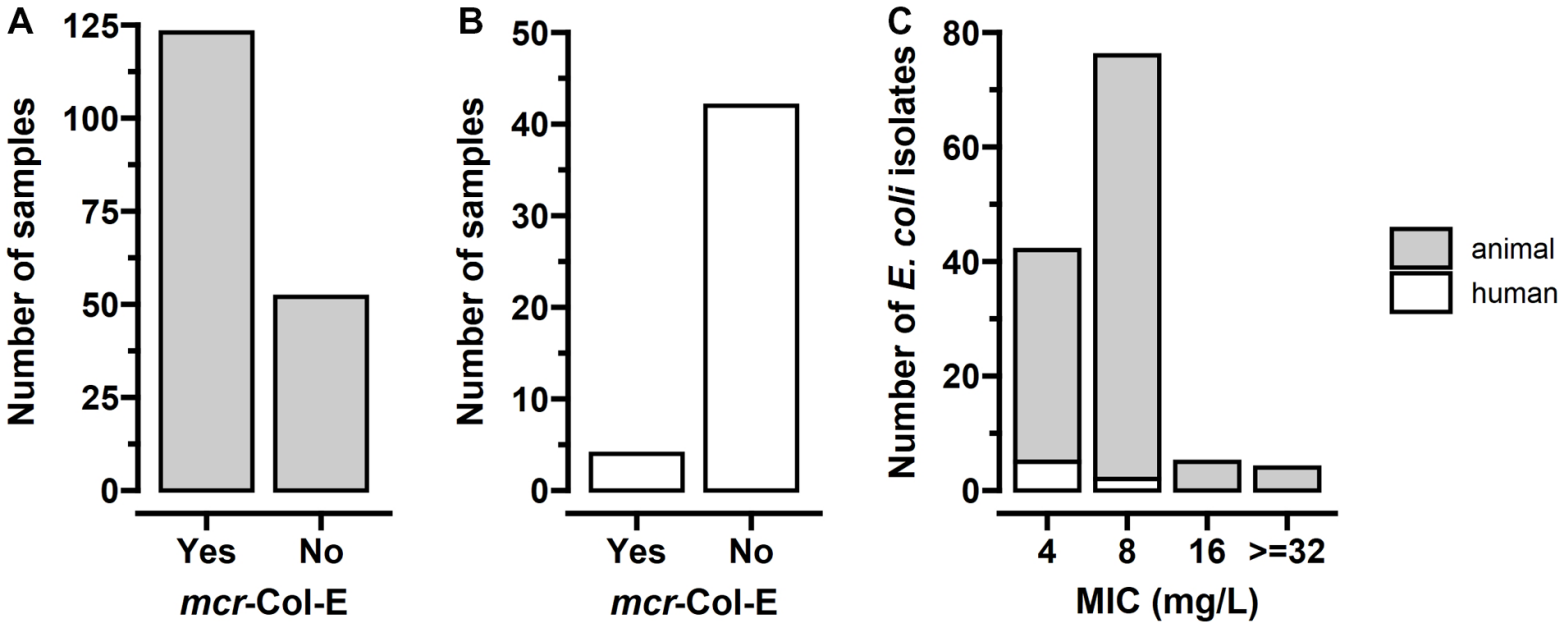
*mcr-*positive colistin-resistant Enterobacterales (*mcr-*Col-E) were highly abundant in the boot swab samples from turkey stables and rare but detectable in human stool samples, with all minimum inhibitory concentrations (MICs) above the epidemiological cut-off value. **(A)** The *mcr-1* gene was present in 123 of the 175 samples from animal faeces (70.3%), harboured by *E. coli* in 121 and *Klebsiella* spp. in two cases (*n* = 175). **(B)** In stool samples from humans, *mcr-*Col-E were present in four samples (8.7%), all *E. coli* (*n* = 46). **(C)** In line with pre-enrichment broth containing 2 mg/l colistin, all MICs were higher than the epidemiological cut-off value of 2 mg/l, with a median MIC of 8 mg/l (*n* = 120 animal and *n* = 7 human isolates). Isolates from turkey stables had higher MICs than those from human stool samples.

The *mcr-1* gene was identified in 123 of the 175 boot swab samples, equivalent to a prevalence of *mcr-*Col-E of 70.3% in the turkey farms included in the study (Figure 1A). Culture of these 123 samples led to 121 *E. coli* isolates (69.1% of all samples and 98.4% of all positive samples). For the remaining two samples, the *mcr-1* gene was harboured by *Klebsiella variicola* and *K. pneumoniae*. The *mcr-2* gene was not detected in any of these samples. After the methods became available, the 141 remaining samples were additionally screened for *mcr-3, mcr-4* and *mcr-5,* and none of them tested positive.

In the 46 human stool samples, seven isolates of *mcr-*Col-E were found. All of them were *E. coli* and stemmed from four different individuals (Figure 1B). One of them harboured three, another person two different isolates, and two participants harboured one isolate each. The two farmers and two family members belonged to two different farms with a conventional husbandry system where *mcr-1*-positive *E. coli* had also been detected in the boot swab samples.

None of these persons had contact with livestock other than poultry, worked in medical care, had taken antibiotics or had been hospitalised within the last 12 months. One person reported an 8- to 10-day-long stay abroad (see Table 2).

There was no connection between colonisation and kind or duration of contact with poultry (dung) (see Table 3).

**Table 3:** Kind or duration of contact (per week) with poultry of participants differentiated by colonisation with *mcr*-Col-E (*n* = 46, not all participants responded to every item).

	*mcr*-Col-E	
	Yes	No
Work with direct contact >10 h	1	27
Work with direct contact <= 10 h	3	15
Cleaning stable >10 h	0	2
Cleaning stable <= 10 h	4	37
Other work inside stable >10 h	0	19
Other work inside stable <= 10 h	4	21
Dung contact outside stable >10 h	0	3
Dung contact outside stable <= 10 h	4	35
Other contact >10 h	0	0
Other contact <= 10 h	3	15

Minimum inhibitory concentrations were measured for only 120 of the 121 animal *E. coli*-isolates because one was overgrown by *Proteus* spp. A MIC of 4 mg/l was found in 37 (30.8%) isolates, 74 (61.7%) had a MIC of 8 mg/l, five (4.2%) of 16 mg/l, and for four (3.3%) the MIC was ≥32 mg/l. Among the isolates from human stool, five (71.4%) had a MIC of 4 mg/l and two (28.6%) of 8 mg/l (Figure 1C).

**Figure 2:**
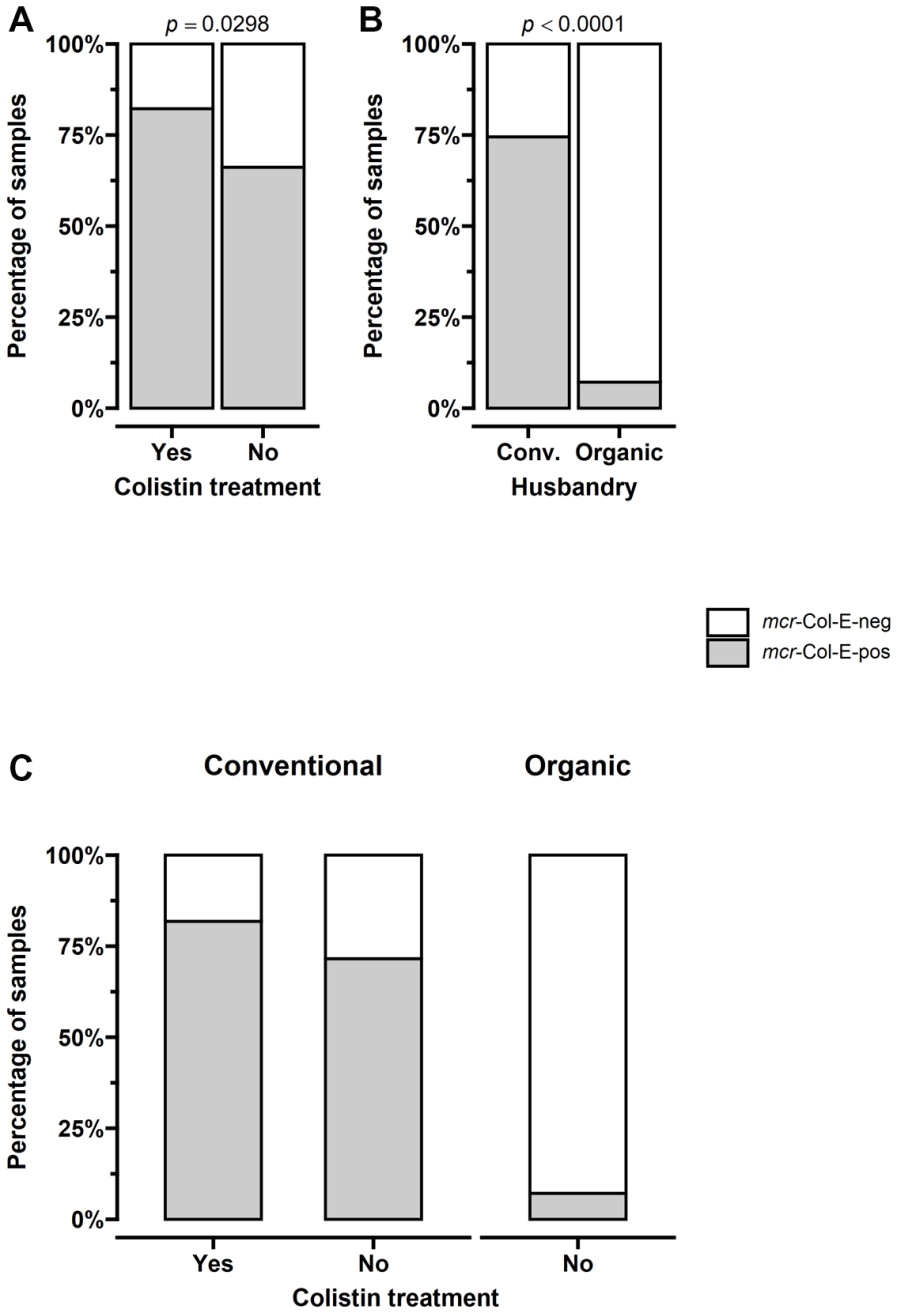
The prevalence of *mcr-*positive Enterobacterales was significantly higher in samples from stables where turkeys had been treated with colistin and in those from conventional farms. **(A)** The prevalence of *mcr-*Col-E was 82.2% (37 of 45) in treated herds and 66.2% (86 of 130) in untreated ones (*p* = 0.0298). **(B)** 74.5% (114 of 153) of conventionally kept herds were *mcr-*Col-E-positive, compared to 7.1% (1 of 14) of organic ones (*p* < 0.001). **(C)** The combination of both factors shows that no organic herd had been treated and that the prevalence in conventional herds was 81.8% (36 of 44) in those that had been treated compared to 71.6% (78 of 109) in those that had not. For **(A)**: *n* = all 175 farms (45 treated, 130 untreated), for **(B,C)**: *n* = 167 farms for which husbandry data was available (153 conventional – 45 treated, 108 untreated- and 14 organic – all untreated).

Detection of *mcr-*Col-E in samples from turkey stables differed depending on whether the animals had been treated with colistin, with a prevalence of 82.2% in the 45 treated herds compared to 66.2% in the 130 untreated ones (*p* = 0.0298). No organic herd had been treated. For one herd where colistin had been administered, there was no data on husbandry. The remaining 44 treated herds were kept under conventional husbandry systems. The prevalence in these conventional treated herds was 81.8% (36 of 44) compared to 71.6% (78 of 109) in conventional herds without colistin treatment. There was also a difference when comparing husbandry systems regardless of treatment: while *mcr-*Col-E were found in 114 of the 153 conventionally kept herds (74.5%), this was only the case for a single one of the 14 organic herds, amounting to 7.1% (*p* < 0.001, Figure 2).

### Carbapenem-resistant Enterobacterales and carbapenem resistance genes

No CPE were detected, neither in the 175 animal-derived samples nor the 46 human stool swabs. Screening for carbapenem resistance genes *bla*_NDM_, *bla*_KPC_, *bla*_VIM_, *bla*_IMP_, and *bla*_OXA-48_ directly in the samples showed that boot swabs from two different farms contained *bla*_OXA-48_ genes. Culturing these samples demonstrated that the *bla*_OXA-48_ genes were harboured by isolates belonging to the non-enterobacterial species *Shewanella* and *Aeromonas*.

### Comparison of colistin-resistant *Escherichia coli* isolates and *mcr-*plasmids

**Figure 3:**
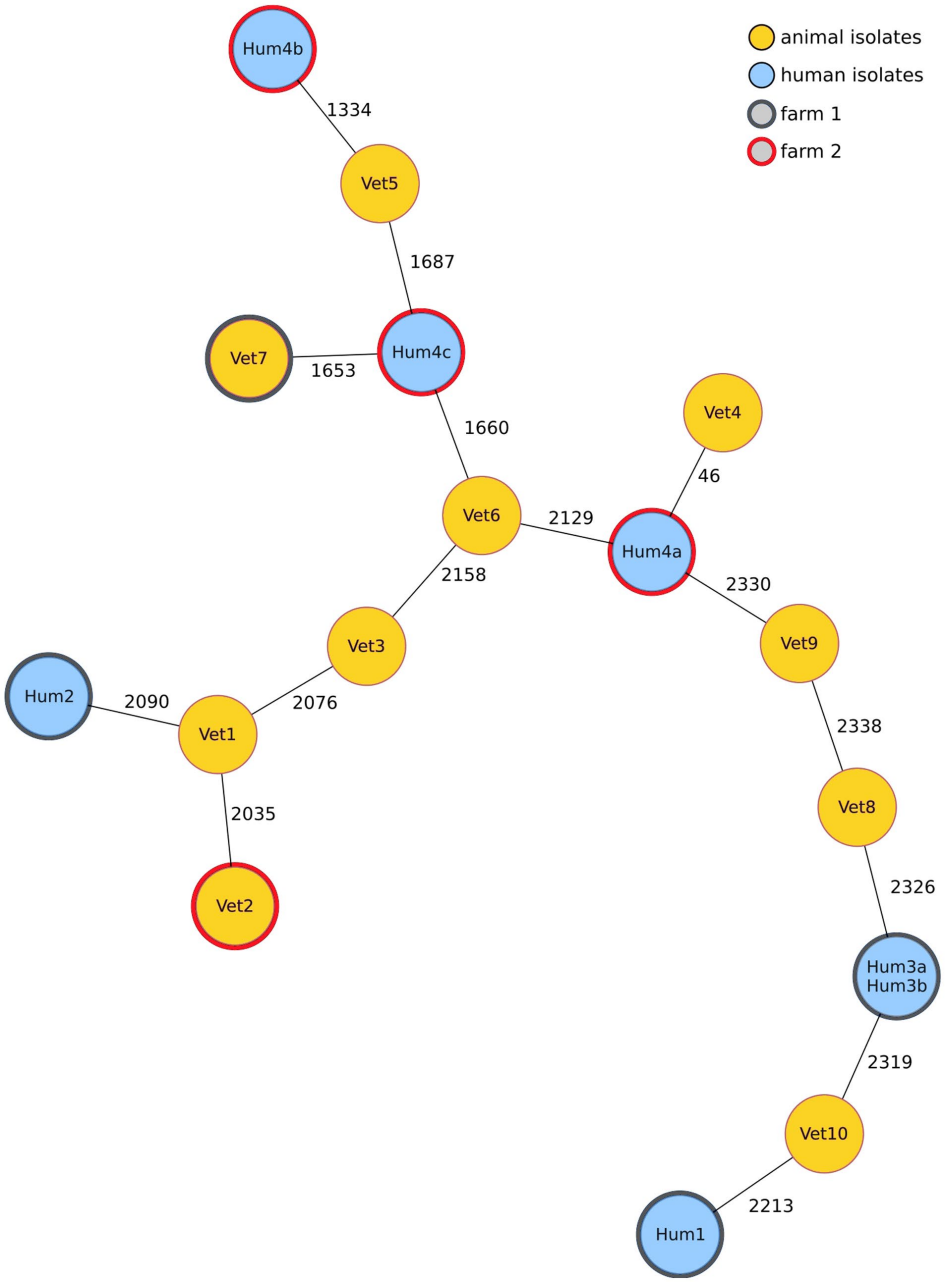
A minimum spanning tree based on the cgMLST allelic profiles of *mcr-1*-positive *E. coli* isolates shows that the two most closely related isolates differed in 46 alleles but originated from different turkey farms. Each node represents an allelic profile based on a sequence analysis of 2,513 cgMLST targets (seven isolates from human participants who lived/worked on farm 1 or 2, the two corresponding veterinary isolates from these farms and eight randomly selected isolates from unrelated farms, sample numbers do not correspond to farm of origin). Missing values were ignored during pairwise comparison. The numbers on the connecting lines correspond to the number of cgMLST targets with different alleles. Node colour depends on the sample origin (animal or human), and isolates with the same border colour are from the same farm.

To find out whether *mcr-1-*positive *E. coli* isolated from turkeys and their human contacts were clonal, suggesting a direct transmission, the isolates were sequenced and compared using cgMLST. Figure 3 shows a minimum spanning tree based on cgMLST allelic profiles of *mcr-1*-positive isolates from 10 turkey farms: two farm isolates and the corresponding seven isolates from human participants on these farms, and eight randomly selected isolates from unrelated farms to investigate the overall diversity of *mcr-1-*positive *E. coli*. Except for two phenotypically different isolates from the same person (Hum3a and Hum3b) which had an identical cgMLST profile, no genotypic clusters were detected. The isolates that were most closely related, Hum4a and Vet4, differed in 46 alleles and originated on different farms. All further isolates differed in more than 1,000 alleles.

To investigate whether a transmission of colistin resistance *via* the exchange of plasmids may have occurred between animal and human isolates, the plasmids carrying the *mcr-1* gene were identified, and their sequences were analysed.

All 17 *mcr-1* plasmids of both environmental and human origin belonged to one of two plasmid family types: one, with a size of ~33–34 kb, harbouring the IncX4 replicon (*n* = 6) and one harbouring IncHI2 family replicons and a size ranging between ~172 kb and 291 kb (*n* = 11). From the latter, four also partially mapped IncQ1 (Table 4).

**Table 4:** Overview of plasmids containing the *mcr-1* gene.

Isolate ID	Farm	Length (bp)	Replicon type
Hum1	1	34,639	IncX4
Hum2	1	172,270	IncHI2, (IncQ1)
Hum3a	1	242,799	IncHI2, (IncQ1)
Hum3b	1	242,799	IncHI2, (IncQ1)
Vet7	1	181,092	IncHI2
Hum4a	2	245,550	IncHI2
Hum4b	2	291,022	IncHI2
Hum4c	2	240,353	IncHI2
Vet2	2	33,303	IncX4
Vet1	\	278,947	IncHI2, (IncQ1)
Vet10	\	34,082	IncX4
Vet3	\	33,310	IncX4
Vet4	\	33,304	IncX4
Vet5	\	33,310	IncX4
Vet6	\	257,485	IncHI2
Vet8	\	217,572	IncHI2
Vet9	\	237,334	IncHI2

Length in bp and detected plasmid replicon types are given for each sequenced isolate (seven isolates from human participants who lived/worked on farm 1 or 2, the two corresponding environmental isolates from these farms and eight randomly selected isolates from unrelated farms).

**Figure 4:**
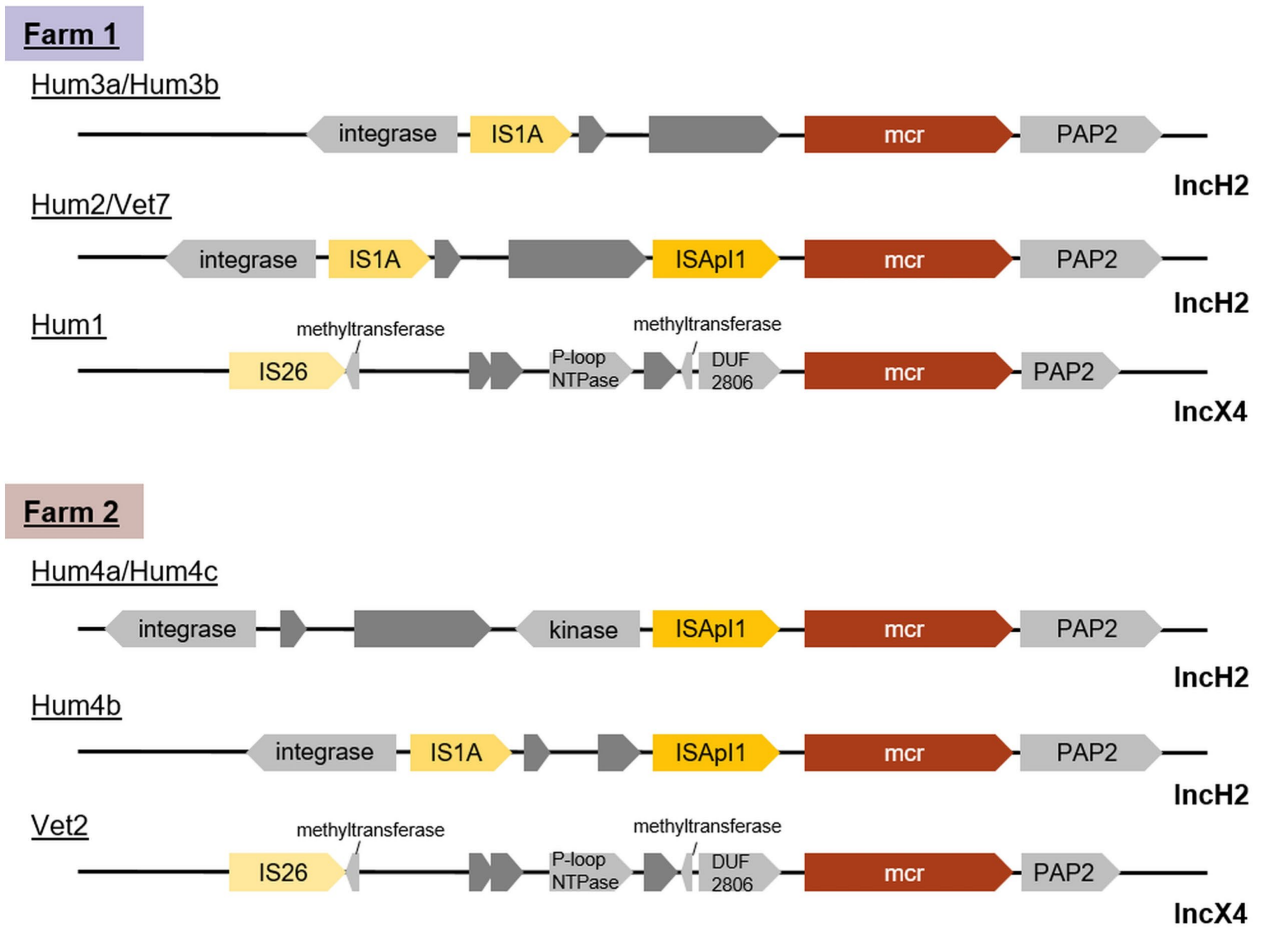
Genetic content of *mcr-1* adjacency on plasmids isolated from two turkey farms shows that the farm-associated isolate Vet7 and the human isolate Hum2 from farm 1 were identical. The *mcr* gene is highlighted in red, transposases in yellow, hypothetical proteins in dark gray and other annotated proteins in light gray.

On farm 2, all human isolates belonged to plasmid type IncHI2 while the animal-associated isolate harboured an IncX4 replicon type plasmid. On farm 1, IncHI2 plasmids were isolated from two persons and the farm environment. A comparison of the region around the *mcr* locus (Figure 4) showed that the human isolate Hum2 and the farm-associated isolate Vet7 were identical. Isolates Hum3a and Hum3b from the same farm were lacking ISApI, an IS30-like family transposase, completely, while all other isolates only lacked a copy downstream of the *mcr-1* cassette. Although isolates Hum4a, Hum4b and Hum4c were taken from the same stool sample and all three harboured IncHI2-type plasmids, they differed in size. Moreover, Hum4b was lacking a kinase close to the *mcr* locus.

## Discussion

This study investigated the prevalence and genomic profiles of *mcr*-Col-E and CPE on German turkey farms as well as among farm personnel using a combination of selective culture, detection of antimicrobial resistance genes, cgMLST and plasmid typing.

Following the initial discovery of *mcr-1* as a mobile resistance gene, subsequent studies aimed to determine the prevalence of the plasmid in colistin-resistant Enterobacterales. Often, these studies retrospectively analysed existing isolates of indicator *E. coli*. These data revealed turkeys as the species most frequently harbouring *mcr-1*-positive isolates in Germany: between 2010 and 2014, the highest overall prevalence, 11.8%, was found in turkeys (faeces taken at farm level and caeca sampled at slaughter). The proportion of *mcr-1-*positive *E. coli* among colistin-resistant isolates ranged from 89.2 to 100% during this time (Irrgang et al., 2016). From 2016 to 2018, Grobbel et al. (2022) found a comparable prevalence of colistin-resistant isolates in faecal samples obtained from conventional turkey farms, at 9%.

However, all these data are based on a statistical population consisting of commensal *E. coli* collected for screening purposes. This method is particularly useful for monitoring time trends in antimicrobial resistance (Hesp et al., 2022). Nevertheless, a more sensitive approach is needed to gain an estimate of the potential spread of *mcr-*Col-E in turkey farming. Although cultural enrichment does not represent clinical impact, it is ideal for identifying all isolates carrying the traits under scrutiny.

Farm level prevalence data based on selective cultural enrichment of *mcr-*Col-E have to date only been available for swine in Germany: Effelsberg et al. (2021) found *mcr-*Col-E in 12.3% of farms and 1.4% of farmers. Klees et al. (2020) used the same methodology and detected *mcr* genes in 43% of samples from a poultry slaughterhouse. However, they were not always able to obtain Col-E isolates from the same samples. As expected, the numbers in the current enrichment-based study are much higher than those that have previously been reported for poultry farms based on screening of commensal *E. coli*: with a prevalence of 70.3% for *mcr*-Col-E in environmental boot swabs of turkey faeces, the results greatly exceed the highest prevalence found by Irrgang et al. (2016), namely 17.9% in faeces at farm level in 2011.

In the present study, all *mcr-1*-positive isolates, with the exception of two, were identified as *E. coli*. These exceptions were *K. variicola* and *K. pneumoniae*, which have previously been shown to harbour the *mcr-1* gene in isolates derived from humans, animals and the environment (Liu et al., 2016; Kieffer et al., 2017; Elbediwi et al., 2019).

The isolates from turkeys in this analysis (*n* = 120) had MICs ranging from 4 to ≥32 mg/l, with a median of 8 mg/l, whereas human isolates (*n* = 7) only ranged from 4 to 8 mg/l. All isolates had been pre-enriched in presence of 2 mg/l colistin. Evidence from other studies is inconclusive: Zając et al. (2019) only reported values of 2, 4, and >4 mg/l in *mcr-1*-positive *E. coli* from food-producing animals, whereas Muktan et al. (2020) observed MICs up to 32 mg/l, with similar ranges in both poultry and human specimens.

Colistin was used in 25.7% of all herds included in the study. *mcr-*Col-E were detected in 80.0% of these herds that had been treated with colistin. Since the beginning of mandatory surveillance of antibiotic use in 2011, veterinary colistin sales in Germany have been reduced by 52.8%, from 127 t to 60 t in 2020 (Bundesamt für Verbraucherschutz und Lebensmittelsicherheit, 2021). However, at 8.6% of all sales, polypeptides are currently the fourth most commonly used group of antibiotics in veterinary medicine, after penicillins, tetracyclines and sulfonamides. It has been shown that a reduction in colistin usage can lead to greatly reduced resistance rates (Zhang et al., 2022), and that complete cessation of colistin use may entirely eliminate Col-E after a transitional period (Randall et al., 2018).

According to the findings of Islam et al. (2020), there is a positive correlation between the frequency of colistin administration in chickens and the prevalence of *mcr* and Col-E. Mesa-Varona et al. (2020) also detected such a relationship for broilers but not for turkeys in Germany. It has been suggested that higher resistance rates in turkeys than in chickens may be due to their longer life span, leading to longer exposure to antimicrobials and therefore higher selective pressure (Zając et al., 2019). Selective pressure of colistin on the intestinal microbiota could be high due to its lack of systemic resorption. In addition, colistin has been reported to often be greatly overdosed in poultry, as highly as 12-fold in chickens (Bundesinstitut für Risikobewertung, 2022; Flor et al., 2022). However, at 66.2%, alarmingly high rates of *mcr-*Col-E were also found in herds that had not been treated with colistin at all. In pigs, Randall et al. (2018) also found a high prevalence of Col-E in untreated animals.

One possible explanation is residual colistin in the stables resulting from past treatments of previously housed herds: colistin has been demonstrated to remain chemically intact for at least 60 days solved in water (Li et al., 2003). Moreover, *mcr*-*1*-carrying IncHI2 plasmids seem to be stable despite their large size (Ma et al., 2018). Plasmids carrying *mcr-1* have likewise been found to remain biologically active even after typical procedures occurring in livestock maintenance such as composting (Le Devendec et al., 2016). Also, the identified plasmids, particularly those of the IncHI2 type, contain a high number of additional antimicrobial resistance genes, potentially adding to a co-selective effect. Furthermore, wild animals may act as a source of Col-E (Franklin et al., 2020). Finally, there could have been cross-transmission of Col-E between herds via bacterial contaminations remaining in the farm environment, or Col-E could have been introduced via poultry that was already colonised when newly transferred to the farm (Majewski et al., 2020). All these circumstances may therefore induce colistin resistance in untreated animals.

Only one of 14 organically farmed turkey herds (7.1%) harboured *mcr-*Col-E, compared to 74.5% of conventionally kept herds. There are no farm-level data on German turkeys for comparison. However, an isolate-level study of commensal *E. coli* detected no colistin resistance in boot swab samples from German organic turkey farms, in contrast to a prevalence of 9% in conventional ones (Grobbel et al., 2022). Like all organically farmed herds in the present study, the *mcr-*Col-E-positive herd had not been treated with colistin, even though colistin is licensed for use in organic turkey farming. On the other hand, prevalence of *mcr-*Col-E was very high (71.6%) in conventional farms without colistin treatment. Therefore, the lack of such treatment alone is not a sufficient explanation. Mughini-Gras et al. (2020) detected higher levels of lysozyme and serum bactericidal activity in organically raised turkeys. As such findings could greatly impact turkey primary production, further studies are needed to determine to what extent the difference can be explained by factors like feed, stocking density and the administration or residues of antibiotics and other substances.

Carbapenemase-producing Enterobacterales were not found in any of the samples examined in this study. Carbapenemases were only detected in oxidase-positive bacteria that have previously been described to harbour *bla*_OXA-48_ (Ceccarelli et al., 2017). In German analyses along the food chain, CPE have only rarely been detected in samples originating from pigs and broilers (Fischer et al., 2012, 2013, 2017; Irrgang et al., 2017, 2020), and the human study population had no risk factors associated with CPE-carriage (Köck et al., 2021).

This study found that 8.7% of the 46 poultry farm workers were carrying *mcr*-Col-E. Studies investigating the prevalence of rectal *mcr*-Col-E carriage of humans in Western European countries observed much lower carriage rates (0.0–0.4%; Terveer et al., 2017; Zurfluh et al., 2017; van Dulm et al., 2019). Contact with livestock is a known risk factor for human colonisation with *mcr-1*-positive bacteria: PCR-screening for *mcr-1* of rectal swabs led to a prevalence of 33.0% in Vietnamese chicken farmers exposed to *mcr-1*-positive chickens, a lot higher than the rates in non-farming participants from rural (17.9%) and urban areas (9.1%; Trung et al., 2017).

It is also possible that the risk is not directly associated with contact with livestock itself because on farms, *mcr*-Col-E have also been isolated from dog faeces, stable flies and manure (Guenther et al., 2017). The colistin treatment of the animals appears to be a contributing factor: Evidence from China suggests that banning the use of colistin as a growth promoter greatly reduced human colonisation by colistin-resistant bacteria (Wang et al., 2020). Similarly, in Thailand, the number of farm workers carrying *mcr-1*-positive *E. coli* dropped from 4 in 10 to 0 after cessation of colistin use in pigs, although detection in pigs and wastewater remained possible even 3 years later (Khine et al., 2022).

It should be highlighted that only one *mcr*-Col-E colonised person in this study reported another risk factor for the acquisition of *mcr*-Col-E except for poultry farm contact, i.e., travel, although it was not assessed whether the travel destination was among those countries associated with an increased risk (Von Wintersdorff et al., 2016; Schaumburg et al., 2019). Underreporting of risk factors was decided to be neglected, as the questionnaire items did not refer to personality traits or require long-term recall. Recently, consumption of fish and seafood has also been suggested as a risk factor in healthy humans (Lv et al., 2022).

Molecular typing revealed the *mcr-1*-positive *E. coli* isolates to be highly diverse, with no clonality between human and animal isolates from the same farms. This corresponds to other findings that also reported great diversity in *mcr-1*-positive *E. coli* (Migura-Garcia et al., 2020; Effelsberg et al., 2021). However, it is a clear limitation of the study design that a maximum of two *mcr*-positive *E. coli* isolates per farm were analysed for a comparison of environmental and human isolates. Given the diversity of *E. coli* prevalent in the farm environment, this makes it unlikely to find matching human-environmental pairs and confirm direct transmission.

However, to improve the comparative techniques, plasmid-typing of *mcr*-Col-E isolates from humans and the farm environment was also performed: the *mcr* gene was contained by two different replicon family types: IncX4 and IncHI2. They have both been found among the most prevalent *mcr*-carrying plasmids not only in Germany (Falgenhauer et al., 2016; Roschanski et al., 2017) and other European countries such as Denmark (Hasman et al., 2015), the Netherlands (Veldman et al., 2016), France (Haenni et al., 2016; Treilles et al., 2022), the United Kingdom (Doumith et al., 2016), Switzerland (Zurfluh et al., 2017), and Spain (Migura-Garcia et al., 2020) but also in Asia (Matamoros et al., 2017). Comparing the plasmids harbouring *mcr* genes from human and environmental isolates from the same farm, there was one case with a similar plasmid backbone and identical *mcr* environment, pointing towards zoonotic transmission. However, considering the lack of clonality of isolates from humans and the farm environment, other risk factors and the technical limitations of this study discussed above, the exact sources for *mcr-*Col-E among the farmers remain unclear.

Besides the limitations already mentioned, further limiting aspects of this study are the following: Due to the use of a more sensitive combination of methods, direct comparison of these turkey prevalence data with other findings, such as those reported by Irrgang et al. (2016), is not possible. Additionally, the study did not include testing for *mcr-6* to *mcr-10* in animal samples or *mcr-3* to *mcr-10* in human samples, as these methods were not available when the protocols were implemented. Also, the study area was limited to Lower Saxony and is hence not representative of Germany. However, Lower Saxony is by far the most significant federal state in the poultry industry: in 2019, 58.5% of turkeys slaughtered in Germany were slaughtered in Lower Saxony (Statistisches Bundesamt (Destatis), 2021), and Lower Saxony regularly provides between 70 to 80% of turkey-associated samples for the national zoonosis monitoring programme, which is based on proportional numbers for all federal states. Additionally, in light of the high prevalence in untreated herds, it would have been desirable to collect data on previous colistin use as well as the use of other antimicrobial agents, even if they had not been applied during the current fattening round. Finally, the number of human participants is rather small. Although various methods of information were used, the motivation on farms to participate in the study was low. This may have been caused not by lack of interest in the study itself but rather by the obstacle of taking a stool sample. Acceptance for taking stool samples can be hard to achieve even for cancer screening (Gordon and Green, 2015). Also, knowledge of colonisation may be perceived as a stigma, and decolonisation is not recommended.

## Conclusion

Reduction and careful evaluation of antibiotic usage must be part of the ongoing efforts in both human and veterinary medicine. This study showed a high prevalence of *mcr-*Col-E on German poultry farms even in herds that had not been treated (66.2%). However, the fact that *mcr-*Col-E were found on only one of 14 organic farms (7.1%) indicates that a combination of several factors may have a favourable influence: These herds not only remained untreated, but were also kept according to other principles of organic farming, such as slower fattening and lower stocking density. Further research is needed to identify the specific contributions of such factors. Overall, carriage of *mcr-*Col-E was 8.7% among farm personnel, which exceeded expectations for the general population. In one case, similar plasmids were found in isolates from the farmer and from environmental samples of the respective farm. However, direct clonal transmission was not detected.

## Data availability statement

The datasets presented in this study can be found in online repositories. The names of the repository/repositories and accession number(s) can be found below: https://www.ncbi.nlm.nih.gov/, PRJNA934726.

## Ethics statement

The studies involving human participants were reviewed and approved by Ethics Committee of the University of Münster (No. 2018-008-f-S). The patients/participants provided their written informed consent to participate in this study.

## Author contributions

DR, KC, JE, and RK: conceptualisation. MS, DR, KC, ACM, AM, IM, CW, JE, and RK: methodology. KN, MS, and NE: formal analysis. KN, MS, NE, CK, DR, KC, RE, AM, IM, SR, CW, AW, and JE: investigation. KN, MS, CK, RE, and JE: data curation. KN and MS: writing – original draft. KN, MS, NE, CK, DR, KC, RE, ACM, AM, IM, SR, CW, AW, JE, and RK: writing – review and editing. DR, KC, JE, and RK: supervision. CK, DR, KC, JE, RK: project administration. DR, ACM, JE, and RK: funding acquisition. All authors contributed to the article and approved the submitted version.

## Funding

This work was funded by the INTERREG Va program of the European Union (project “EurHealth-1Health,” grant number EU/INTERREG VA-681377) and by the German Federal Ministry for Education and Research (BMBF) within the Research Network Zoonotic Infectious Diseases (project #1Health-PREVENT, grant numbers 01KI1727A, 01KI2009A). We further acknowledge support by the Deutsche Forschungsgemeinschaft (DFG, German Research Foundation) – project 281125614/GRK2220. For the publication fee, we acknowledge financial support by Deutsche Forschungsgemeinschaft within the funding programme “Open Access Publikationskosten” as well as by Carl von Ossietzky University of Oldenburg. The funders had no role in study design, data collection and interpretation, or the decision to submit the work for publication.

## Conflict of interest

The authors declare that the research was conducted in the absence of any commercial or financial relationships that could be construed as a potential conflict of interest.

## Publisher’s note

All claims expressed in this article are solely those of the authors and do not necessarily represent those of their affiliated organizations, or those of the publisher, the editors and the reviewers. Any product that may be evaluated in this article, or claim that may be made by its manufacturer, is not guaranteed or endorsed by the publisher.

## Footnotes

^1^ http://enterobase.warwick.ac.uk
